# Outcomes of post-operative drain use after cranioplasty surgery – a systematic review and meta-analysis

**DOI:** 10.1007/s00701-025-06766-3

**Published:** 2026-01-13

**Authors:** Zhikai Li, Yuhan Guo, Shangqing W. Yang, Munashe Veremu, Youssef Chedid, William H. Cook, Mohammad Ashraf, Matthew Kingham, Alexandra Lisitsyna, Marwan Al-Munaer, Keng Siang Lee, Harry Mee, Yevgeny Karepov, Conor S. Gillespie, Adel Helmy, Ivan Timofeev, Peter J. Hutchinson

**Affiliations:** 1 Department of Clinical Neurosciences, Addenbrooke’s Hospital, Cambridge, UK; 2 Department of Neurosurgery, Addenbrookes’s Hospital, Cambridge, UK; 3 Wolfson School of Medicine, University of Glasgow, Glasgow, Scotland, UK; 4 Imperial College London Medical School, London, UK; 5 Department of Neurosurgery, King’s College Hospital, London, UK; 6 Department of Basic and Clinical Neurosciences, Maurice Wohl Clinical Neuroscience Institute, Institute of Psychiatry, Psychology and Neuroscience (IoPPN), King’s College London, London, UK; 7 University of Cambridge, Cambridge, UK; 8 School of Clinical Medicine, University of Cambridge, Cambridge, UK

**Keywords:** Cranioplasty, Subgaleal drain, Epidural drain, Post-operative complications

## Abstract

**Background:**

Cranioplasty restores cranial integrity following decompressive craniectomy or skull trauma. Despite its reconstructive benefits, post-cranioplasty complication rates are high. Post-operative drainage has been proposed to mitigate these risks, yet its effectiveness remains uncertain. This study evaluates the impact of post-cranioplasty drain insertion on surgical outcomes.

**Methods:**

A systematic literature search of MEDLINE, Embase, and Cochrane CENTRAL Library was conducted in accordance with PRISMA guidelines (PROSPEROID:CRD420251030365). Studies reporting cranioplasty outcomes with post-operative drainage were selected. Primary outcomes were complication rates, including infection, haemorrhage, and cerebrospinal fluid (CSF) leak.

**Results:**

Four studies met the inclusion criteria, comprising 522 patients (mean age 43.7 years) who underwent cranioplasty—282 with post-operative drainage and 240 without. Following decompressive craniectomy, the most common indications for cranioplasty were traumatic brain injury (196/514, 38.1%), vascular causes (187/514, 36.4%), and infection (25/514, 4.9%). All studies reported subgaleal drain use, with one study (25%) using epidural drains in an unspecified number of patients. The overall post-operative complication rate was 75/522 (14.4%), occurring in 23/282 drained patients (8.2%) and 52/240 (21.7%) undrained patients. A meta-analysis comparing post-operative complication rates across all studies between patients with and without post-cranioplasty drainage yielded a pooled risk ratio (RR) of 0.51 (95% CI: 0.21–1.24, *p* = 0.095).

**Conclusions:**

The results suggest post-cranioplasty drainage does not significantly alter complication rates. However, heterogeneity in drainage protocols limits attribution of outcomes to specific modalities. Going forward, moderated prospective trials are needed to establish standardised post-cranioplasty drainage protocols.

**Supplementary Information:**

The online version contains supplementary material available at 10.1007/s00701-025-06766-3.

## Introduction

Cranioplasty is a neurosurgical procedure aimed at restoring cranial contour and cerebral protection following decompressive craniectomy or traumatic skull injury [15, 23, 25, 34]. Despite its reconstructive benefits, significant post-operative complications can arise, including infection (estimated 12.3–29.7% risk), CSF leak (estimated risk of 4.5–14.3%), haemorrhage (estimated ~ 12% risk), and bone resorption [4, 8, 11, 18, 20, 26, 32, 35, 37], all of which impact functional recovery and long-term patient outcomes.

Post-operative drainage has been suggested to reduce fluid accumulation, prevent wound-related complications, and optimise recovery [1, 12, 14]. Subgaleal and epidural drainage can be employed to evacuate blood and serous collections in the immediate post-operative period [5, 13, 18]. However, meticulous haemostasis [2, 5, 27], watertight closure, and dead‑space obliteration [31] are also routinely employed intra-operatively to minimise post‑operative collections, raising the question of whether drains are always necessary. While some studies suggest these drainage techniques improve post-operative outcomes, conflicting concerns exist regarding infection risk, over-drainage, and potential pressure-related complications such as paradoxical herniation from excessive negative pressure or impaired cerebral perfusion from rapid intracranial decompression [3, 7, 33, 36].

Evidence regarding the comparative efficacy of post-operative drainage methods remains limited, with inconsistencies in reporting protocols, follow-up durations, and drainage implementation strategies across studies. Whether and why patients receive post-operative drainage is also not well documented [19, 29] and standardisation of decision making in this regard would ensure a more consistent and well-informed standard of care for patients. This review aims to provide a systematic evaluation of comparative outcomes between cohorts receiving post-operative drainage compared to no drainage following cranioplasty, focusing on key surgical complications, including infection, CSF leakage, haemorrhage, and bone resorption, to better inform clinical practice.

## Methods

This systematic review was conducted in accordance with the Preferred Reporting Items for Systematic Reviews and Meta-Analyses (PRISMA) statement [22]. The review protocol was prospectively registered on PROSPERO (Registration Number: CRD420251030365).

### Information sources

MEDLINE, Embase, and the Cochrane Library were queried on 11th April 2025 for all studies published since inception. Search strategies for each database were developed through an iterative process and are available in Supplementary Table 1.

### Eligibility criteria

Studies comprising patients who underwent cranioplasty with routine post-operative drainage, irrespective of cranioplasty material or type of drainage device were included. Only studies reporting relevant outcomes (overall survival, complication rate, time to recovery) were included. Studies were excluded if outcomes could not be extracted, or if there was no undrained cohort. Editorial letters, conference abstracts/posters/proceedings, case reports and series with < 5 patients, and systematic reviews were also excluded [16]. Only studies published in English were considered.

### Selection process

All records were imported into Rayyan (Rayyan Systems Inc, Cambridge, MA, USA) [21] for de-duplication, and for abstract and full-text screening. Three authors (ZL, SWY and YG) independently assessed titles and abstracts for eligibility. Full texts of potential articles were then evaluated independently by the same authors. Remaining conflicts were subsequently resolved by discussion amongst authors; if no consensus was reached, a senior author (CSG) made the final decision, and studies were included only if consensus was reached amongst all reviewers.

### Data extraction and synthesis

Data was manually extracted by three authors (ZL, SWY and YG) using a standardised Microsoft Excel spreadsheet. Extracted variables included: author(s), year of publication, baseline demographics, sample size, cranioplasty detail (indication, site, material, surgical detail), drain type and duration, complications (infection, bleeding, bone flap resorption, and any others); methods for complication rectification; mean and median hospital stay durations; and overall outcomes at different time points (1, 3, 6 and 12 months) following cranioplasty. Infection was defined as cranioplasty surgical site infection as reported by the original study authors, not including post-operative infections without cranioplasty site involvement. No automation tools were used to extract data. The primary objective is to present the complication rates following drainage use post-cranioplasty. Random-effects meta-analysis was then used to compare complication rates between drain and non-drain groups, using established methods [9, 17].

### Risk of bias assessment

Risk of Bias was assessed independently using the Newcastle–Ottawa Scale for cohort studies [30] by two reviewers (ZL and SWY) before independent cross-checking with at least one other reviewer.

## Results

### Study selection

**Fig. 1:**
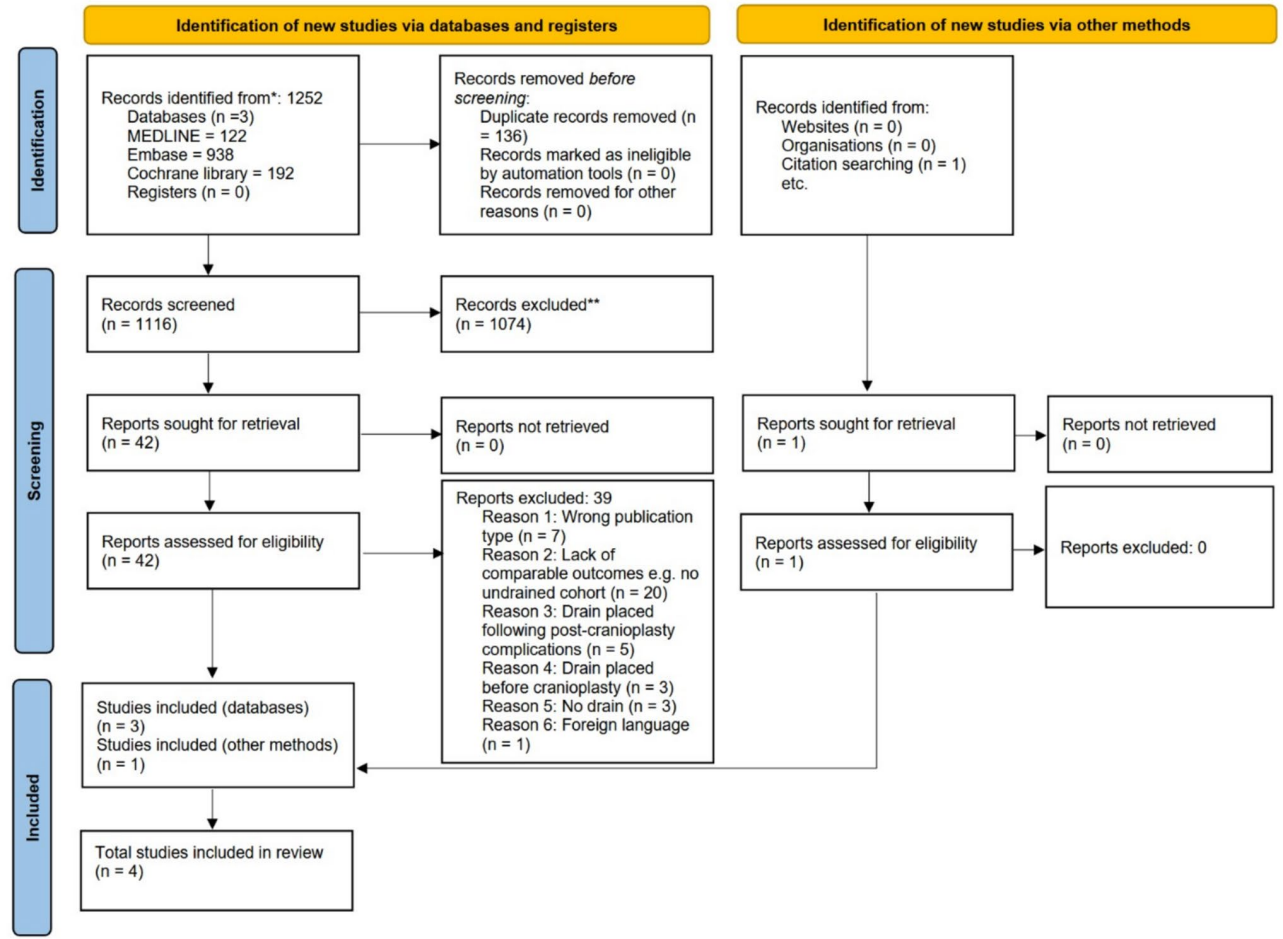
PRISMA flowchart

A total of 1,252 studies reporting outcomes following cranioplasty with post-operative drainage were identified through our search: 122 from MEDLINE, 938 from Embase and 192 from the Cochrane Library. After removal of duplicates and screening for titles and abstracts, full text reviews were performed on 42 studies. Of these, 3 studies met the inclusion criteria and were included in the review, with one additional study identified from citation searching (Fig. 1).

### Study characteristics

Study characteristics are outlined in Table 1. Of the 4 studies, all (100%) were retrospective cohort studies with a total of 522 patients who underwent cranioplasty. Sex was reported in three (75%) studies, within which there were 271/434 males (62.4%) and 163/434 females (37.6%).

**Table 1:** Study characteristics

Author (year)	Design	Population that underwent cranioplasty	Mean age (SD)	Female: Male (%)	% Indication for cranioplasty (*n*)
Spake et al. [29]	Retrospective single-centre cohort	126	44 (19)	46: 80 (37: 63)	56% TBI (71), 2% Neoplasm (3), 38% Vascular (48), 3% Other (4)
Liang et al. [19]	Retrospective single-centre cohort	88	36.2 (range 16–74)	NR	52% TBI (46), 11% Infection (10), 15% Vascular (13), 9% Neoplasm (8), 2% Oedema (2), 10% Other (9)
Sobani et al. [28]	Retrospective single-centre cohort	96	33 (15)	26: 70 (27: 73%)	57% TBI (55), 24% Vascular (23) 10% Neoplasm (10), 8% NR (8)
Chang et al. [6]	Retrospective single-centre cohort	212	43.7 (15.9)	121: 91 (57: 43%)	37% TBI (79), 49% Vascular (103), 6% Neoplasm (13), 7% Infection (15), 1% Other (2)

Baseline characteristics were not stratified by drainage status, drainage stratified outcomes therefore not available. Abbreviations: *NR* Not Reported, *SD* Standard Deviation, *TBI* Traumatic Brain Injury

### Indication for cranioplasty

The most common indications for cranioplasty were traumatic brain injury (TBI) (251/514, 48.8%), vascular (187/514, 36.4%), and infection (25/514, 4.9%). The full range of indications and their relative frequencies are shown in Table 2.

**Table 2:** Cranioplasty indications

Indication	Number of patients (%)
TBI	251 (48.8%)
Vascular	187 (36.4%)
Infection	25 (4.9)
Neoplasm	34 (6.6%)
Oedema	2 (0.4%)
Other	15 (2.9%)

### Cranioplasty procedural characteristics and post-operative drain usage

Procedural characteristics are summarised in Table 3. All studies (100%) reported cranioplasty material and drain use. Autologous bone was the most common material used (420/522, 80.5%). Post-operative drainage was employed in 282/522 (54.0%) of patients, with subgaleal drainage reported in all studies (100%). One study (25%) reported use of epidural drains without specifying exact numbers. Two papers (50%) reported duration of drain placement, ranging from 1 to 3 days on average (Table 3). Complications and outcomes associated with drainage are summarised in Table 4.

**Table 3:** Procedural characteristics

Author (year)	% cranioplasty material	Time to cranioplasty	Length of cranioplasty surgery (minutes)	Additional Intervention Intra-operatively	% Drain (n)	Duration of drain
Spake et al. [29]	100% Autologous (126)	Mean (SD): 108 (81) days	Mean (SD): 200 (5)	NR	78% Subgaleal (98)	Mean (SD): 3 (1.6) days
Liang et al. [19]	61% Autologous (54), 19% Titanium (17), 1% Acrylic and Titanium (1), 10% Acrylic (9), 8% PEEK (7)	56% < 6 months (*n* = 49), 44% > = 6 months (*n* = 39)	Mean (range): 95 (30–200)	NR	83% Subgaleal (73)	1–2 days
Sobani et al. [28]	68% Autologous bone (65), 16% PMMA (15), 11% PMMA + autologous bone (11), 5% Bone flap autoclaved (5)	Mean (SD): 90 (116) days	Mean (SD): 185.2 (67.3)	18% Intra-operative Blood Transfusions (n = 17)	28% Subgaleal (27)	NR
Chang et al. [6]	83% Autologous bone (175), 17% Non-autologous bone (37)	Mean (SD): 5.35 (4.85) months	NR	NR	40% Subgaleal and/or epidural (84)	NR

*NR* Not reported, *PEEK* Polyetheretherketone, *PMMA* Poly(Methyl methacrylate)

**Table 4:** Complications and outcomes associated with drainage

Author (year)	Infection		Other complications*	
	Drain	No drain	Drain	No drain
Spake et al. [29]	4	6	NR	NR
Liang et al. [19]	1	0	NR	0**
Sobani et al. [28]	NR	NR	7	27
Chang et al. [6]	NR	NR	9	25

Infection was variably defined across studies. Non cranioplasty site infections were excluded

*Complications not stratified in Sobani et al. [28] and Chang et al. [6] included bone resorption, fluid accumulation, seizures, and osteomyelitis

**No complications occurred in the undrained group in Liang et al. [19]

### Outcomes

**Fig. 2:**
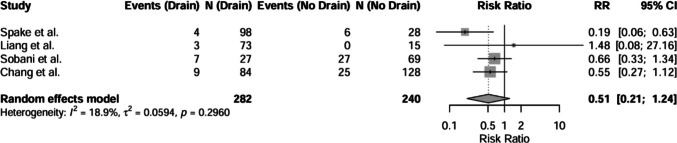
Forest plot showing relative risk ratio (95% confidence interval) for complication rate between drained and undrained groups. Grey squares indicate study weights, with larger squares representing greater influence. The diamond represents the overall pooled effect. Continuity corrections were applied to zero events. Abbreviations: CI = confidence interval; RR = Risk Ratio

The pooled complication rate between drained and undrained post-cranioplasty patients was compared by meta-analysis (Fig. 2), which revealed a lower complication rate in the drain group, with a random-effects risk ratio (RR) of 0.51 (95% CI: 0.21–1.24, I^2^ = 18.9%, *p* = 0.095). Individual study RRs ranged from 0.19 [29] to 1.48 [19]. The largest weights were contributed by Sobani et al. [28] (39.9%) and Chang et al. [6] (39.3%), both of which reported RRs < 1.

**Fig. 3:**
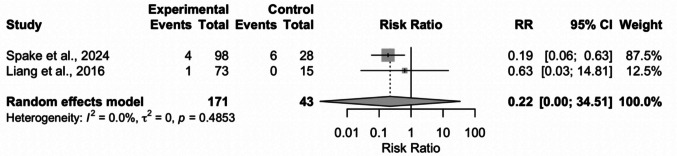
Forest plot showing relative risk ratio (95% confidence interval) for infection rate between drained and undrained groups. Grey squares indicate study weights, with larger squares representing greater influence. The inverse variance method was used. The diamond represents the overall pooled effect. Continuity corrections were applied to zero events. Abbreviations: CI = confidence interval; RR = Risk Ratio

A separate meta-analysis (Fig. 3) was performed to assess post-operative infection rates between drained and undrained cranioplasty patients which were only reported in two studies [19, 29]. The pooled random-effects risk ratio (RR) was 0.22 (95% CI: 0.0014–34.51, *p* = 0.164), indicating no statistically significant difference in infection risk between groups. Individual study estimates varied widely, with Spake et al. reporting a RR of 0.19 (95% CI: 0.06–0.63) and Liang et al. reporting a RR of 0.63 (95% CI: 0.03–14.81). Heterogeneity was very low (I^2^ = 0.0%, τ^2^ = 0), and the Hartung-Knapp adjusted model was used to account for small-study effects. These findings suggest that while drainage may reduce overall complication rates, there is currently no robust evidence linking post-operative drainage to decreased infection risk.

Other reported complications included seizures [6, 28, 29], hydrocephalus [29], and non-infectious wound site complications such as dehiscence resulting from hardware exposure [29] and poor wound healing [19] amongst others. These were not differentiated between drained and undrained patients.

### Risk of bias

Three studies were classified as ‘good’ quality according to the Agency for Healthcare Research and Quality (AHRQ) standards, demonstrating robust methodological design and adequate follow-up. These studies consistently scored well across the Newcastle–Ottawa Scale (NOS) domains, with representative cohorts, secure exposure ascertainment, and comprehensive outcome assessment. One study was rated ‘poor’ due to limited cohort representativeness, unclear selection of the comparison group, and insufficient follow-up reporting. Full scoring details are provided in Supplementary Table 2.

## Discussion

### Key findings

This systematic review and meta-analysis evaluated the impact of post-cranioplasty drainage on surgical outcomes, comparing complication rates between patients with and without subgaleal drain placement. Across four retrospective cohort studies comprising 522 patients, post-operative drainage was associated with a lower pooled complication rate compared with no drainage (8.2% vs 21.7%), yielding a risk ratio of 0.51 (95% CI: 0.21–1.24) [6, 19, 28, 29]. A separate meta-analysis of post-operative infection rates, limited to two studies [19, 29], also found no statistically significant difference between drained and undrained groups (random-effects RR = 0.22, 95% CI: 0.0014–34.51, *p* = 0.164). Although individual estimates favoured drainage, the wide confidence intervals and small sample size preclude firm conclusions. These findings suggest that while post-cranioplasty drainage may reduce overall complication rates and infection, there is currently insufficient evidence to support a protective effect against complications.

### Study consistency and heterogeneity

The low heterogeneity observed (I^2^ = 18.9%) suggests consistency across studies despite differences in sample size, drain protocols, and complication definitions. Notably, the largest contributors to the pooled estimate—Sobani et al. [28] and Chang et al. [6]—both demonstrated reduced complication rates in the drain group.

In contrast, Liang et al. [19] reported a higher complication rate with drainage, finding no significant association between drain use and complications on chi-squared analysis, attributing this to the class imbalance caused by low numbers of undrained patients. This methodological limitation likely explains the disparity observed between their result and the other included studies.

Subgaleal drainage was the predominant modality across all studies, with limited reporting on epidural drain use [6] and no stratification by drain type, preventing modality specific analysis of drainage outcomes. Furthermore, complications were variably defined and inconsistently stratified: some studies reported only infection rates [19, 29], whilst others included fluid collections, haematomas, or reoperations without clear attribution for example to drained and undrained cohorts [6, 28].

### Possible mechanisms of benefit

The observed reduction in complications with drainage may reflect improved evacuation of post-operative fluid collections and reduced wound tension [10, 24, 38].

These findings support the hypothesis that subgaleal drainage may reduce post-operative complications following cranioplasty. However, the evidence remains inconclusive. Future prospective studies should incorporate stratified drain protocols, standardised outcome definitions, and longer follow-up durations to clarify the role of drainage in post-operative fluid management.

### Limitations

This review is subject to several limitations. All included studies were retrospective, and none employed randomisation or adjusted for baseline differences between drain and no-drain groups. Complication definitions and reporting was highly variable: while infection was reported, this was variably defined and inconsistently characterised. Other outcomes such as CSF leakage, haemorrhage, bone resorption, wound healing problems, re‑operation rate, bone flap survival, and hospital stay were inconsistently described and never stratified by drainage status, preventing comparative analysis. Drainage protocols were also inconsistently detailed, with limited information on drain type, placement technique, duration, and removal criteria, limiting generalisability. As drainage was almost exclusively subgaleal, with only one study including epidural drains without stratification [6], modality‑specific analysis was not possible.

Follow‑up durations were short or not reported, restricting assessment of delayed complications. Long‑term functional and patient‑reported outcomes were absent, and it was unclear why drains were left in situ in some cases and removed in others. These factors collectively limit the strength of conclusions and highlight the need for prospective, standardised studies with consistent outcome definitions and stratification by drainage status.

### Future directions

To clarify the role of post-operative drainage in cranioplasty, future research should prioritise prospective, multicentre cohort studies or randomised controlled trials with standardised drainage protocols. These studies should stratify patients by drain type, duration, and cranioplasty material, and control for key confounders such as age, indication for surgery, and comorbidities.

Uniform definitions of complications and consistent reporting of outcomes—including infection, CSF leak, haemorrhage, and fluid collections—will be essential for meaningful comparison. Extended follow-up periods should be incorporated to capture late complications and assess long-term recovery, which was rarely reported in existing studies. Additional inclusion of functional outcomes, patient satisfaction, and cost-effectiveness metrics will further enhance the clinical relevance of future studies.

Importantly, these results suggest a lower complication rate in patients with drains, supporting the link between effective removal of post-operative fluid and reduced risk of adverse events. This highlights a potential key role for appropriate drainage during post-operative fluid management. Ultimately, establishing evidence-based post-operative fluid management strategies will require collaborative efforts across institutions to standardise protocols and improve the quality of data available for analysis.

## Conclusion

This systematic review and meta-analysis suggest that subgaleal drainage following cranioplasty may be associated with a lower post-operative complication rate compared to no drainage, with a pooled risk ratio of 0.51. Although statistical significance was not reached, the trend towards reduced complications and low heterogeneity across studies supports the potential benefit of drainage. However, limitations in study design, inconsistent outcome reporting, and lack of standardised drainage protocols constrain definitive conclusions. Future prospective studies with stratified drain modalities and uniform complication definitions are needed to establish evidence-based post-operative fluid management strategies in cranioplasty.

## Supplementary Information

Below is the link to the electronic supplementary material.

ESM 1 Supplementary Material 1 (DOCX 14.9 KB)

## Data Availability

No datasets were generated or analysed during the current study.
