# Supplementary material for: Outcomes of post-operative drain use after cranioplasty surgery – a systematic review and meta-analysis
Source: Acta Neurochir (Wien). 2026 Jan 13;168(1):10. doi: 10.1007/s00701-025-06766-3 (PMC12804277; doi:10.1007/s00701-025-06766-3)
Supplement: Supplementary file 1 — Supplementary Material 1 (DOCX 14.9 KB) [file 701_2025_6766_MOESM1_ESM.docx]

**Supplementary Table 1.** Search strategy

| Database | No | Search Query | Results |
| --- | --- | --- | --- |
| MEDLINE | | | |
|  | #1 | (Cranioplasty.mp. OR (Cranioplast* OR autologous* OR bone flap* OR synthetic*).mp.)  AND  ((Subgaleal Drain* OR Lumbar Drain*).mp. OR ((Subgaleal* OR lumbar* OR drain* OR subgaleal) ADJ drain*).ab.kf.kw.ti.)  AND  (Outcome* OR infect* OR haemorrhag* OR complicat* OR bone resorp* OR leak*)  Limits applied: Humans, English language, publication types including case reports, clinical studies, randomized controlled trials, and observational studies. | 122 |
| Embase | | | |
|  | #1 | (Cranioplasty.mp. OR (Cranioplast* OR autologous* OR bone flap* OR synthetic*).mp.)  AND  (exp drain/ OR ((Subgaleal* OR lumbar* OR drain* OR subgaleal) ADJ drain*).ab,kw,kf,ti.)  AND  (Outcome* OR infect* OR haemorrhag* OR complicat* OR bone resorp* OR leak*).mp.  Limits applied: Human studies, English language, selected publication types including clinical trials, observational studies, and technical reports. | 938 |
| Cochrane Library | | | |
|  | #1 | (Cranioplasty OR Cranioplast* OR autologous* OR bone flap* OR synthetic*)  AND  (Subgaleal drain* OR Lumbar drain* OR Subgaleal* OR lumbar* OR drain* OR subgaleal ADJ drain*)  AND  (Outcome* OR infect* OR haemorrhag* OR complicat* OR bone resorp* OR leak*)  Filters applied: English language, human studies, relevant publication types. | 769 |

**Supplementary Table 2.** Newcastle-Ottawa assessment of risk of bias for the included studies.

| **Table S2. Paper Title (Cohort Studies)** | Representativeness of exposed cohort | Selection of nonexposed cohort | Ascertainment of exposure | Demonstration outcome of interest not present at start of study | Comparability of cohorts on the basis of the design or analysis controlled for confounders | Assessment of outcome | Follow-up long enough for outcomes to occur (up to 4 as judged by ZL) | Median duration of FU in months and brief rationale for prior assessment | Adequacy of FU cohorts | AHRQ standard conversion* |
| --- | --- | --- | --- | --- | --- | --- | --- | --- | --- | --- |
| Subgaleal drains may be associated with decreased infection following autologous cranioplasty: a retrospective analysis | c | c | a | a | a | b | b | NS | d | P |
| Cranioplasty outcomes and associated complications: A single-centre observational study | b | c | a | a | a | b | a | 3 | a | G |
| Cranioplasty after decompressive craniectomy: An institutional audit and analysis of factors related to complications. | b | c | a | a | a | b | b | NS | a | G |
| Outcomes of cranial repair after craniectomy | b | c | a | a | a | b | a | a | a | G |

* Study quality was rated using the Newcastle-Ottawa Scale (NOS) and mapped to AHRQ standards: *good*—3–4 stars in selection, 1–2 in comparability, 2–3 in outcome/exposure; *fair*—2 in selection, 1–2 in comparability, 2–3 in outcome/exposure; *poor*—0–1 in selection, 0 in comparability, or 0–1 in outcome/exposure.
